# Hearing Preservation after Cochlear Implantation: UNICAMP Outcomes

**DOI:** 10.1155/2013/107186

**Published:** 2013-03-17

**Authors:** Guilherme Machado de Carvalho, Alexandre C. Guimaraes, Alexandre S. M. Duarte, Eder B. Muranaka, Marcelo N. Soki, Renata S. Zanotello Martins, Walter A. Bianchini, Jorge R. Paschoal, Arthur M. Castilho

**Affiliations:** Otology, Audiology and Implantable Ear Prostheses, Ear, Nose, Throat and Head & Neck Surgery Department, P.O. Box 6111, Campinas University, UNICAMP, 13081-970 São Paulo, SP, Brazil

## Abstract

*Background*. Electric-acoustic stimulation (EAS) is an excellent choice for people with residual hearing in low frequencies but not high frequencies and who derive insufficient benefit from hearing aids. For EAS to be effective, subjects' residual hearing must be preserved during cochlear implant (CI) surgery. *Methods*. We implanted 6 subjects with a CI. We used a special surgical technique and an electrode designed to be atraumatic. Subjects' rates of residual hearing preservation were measured 3 times postoperatively, lastly after at least a year of implant experience. Subjects' aided speech perception was tested pre- and postoperatively with a sentence test in quiet. Subjects' subjective responses assessed after a year of EAS or CI experience. *Results*. 4 subjects had total or partial residual hearing preservation; 2 subjects had total residual hearing loss. All subjects' hearing and speech perception benefited from cochlear implantation. CI diminished or eliminated tinnitus in all 4 subjects who had it preoperatively. 5 subjects reported great satisfaction with their new device. *Conclusions*. When we have more experience with our surgical technique we are confident we will be able to report increased rates of residual hearing preservation. Hopefully, our study will raise the profile of EAS in Brazil and Latin/South America.

## 1. Introduction

Just over a decade ago people with sensorineural hearing loss had 2 main hearing (re)habilitation options: (1) a hearing aid (HA) if they had mild to moderate hearing loss and (2) a cochlear implant (CI) if they had severe to profound hearing loss. These 2 device options improved most users' hearing. However, people who could hear in the low frequencies (up to 1000 Hz) but not the medium and high frequencies—the downward or “ski slope” audiogram—had too much high frequency hearing loss to benefit from their hearing aid(s) but were not CI candidates because surgeons feared the surgery would destroy their residual hearing.

A solution for such people is electric-acoustic stimulation (EAS), a concept developed by von Ilberg and colleagues in 1999 [1]. EAS provides synergistic unilateral acoustic (via the HA) and electrical (via the CI) stimulation and provides its users with better hearing than they had had with their HA or HAs [2–4] and better hearing than enjoyed by unilateral CI-only users [1–5], especially in noisy environments [2–4, 6–9]. EAS also provides better sound quality and more natural hearing than unilateral CIs or HAs [4, 10]. These benefits are, however, only possible if surgeons do not damage the cochlea (and thus the person's residual hearing) during CI surgery. To this end, technology and “soft surgical” techniques have been—and are continuing to be—developed.

“Soft” surgery was first described by Lehnhardt and Laszig in 1994 [11] and multiple surgeons and their teams have since refined it [12–16]. Electrode insertion is of utmost importance in atraumicity: the round window approach [15] has shown to cause minimal cochlear damage and is thus better for residual hearing preservation [4, 10].

Electrode design (shape, length, and bundle flexibility) is also critical to reducing cochlear trauma [17–21]; both MED-EL (Innsbruck, Austria) and Cochlear Limited (Sydney, Australia) have designed electrodes to meet this specific need. Focusing on MED-EL's FLEX^24^ (formerly known as the FLEX^EAS^), as it is the electrode we used in our study, recently surgeons have used it to achieve partial or complete hearing preservation in 100% of their study subjects [10, 16]. Although such perfection is not always possible, regardless of the surgeon's skill or the implanted device's technical wizardry [22], it was our aim to preserve the residual hearing in each of the 6 subjects we implanted between March 2010 and October 2011. 

## 2. Materials and Methods

### 2.1. Subjects

6 subjects (mean age 47 years) were implanted with MED-EL cochlear implants with FLEX^24^ electrodes. Study inclusion criteria were as follows: all subjects had to (1) be older than 18 years, (2) have sensorineural bilateral hearing loss with little or no benefit from HA (less than 40% of auditory discrimination in monosyllables), (3) have pure-tone thresholds of ≤60 dB hearing loss in at least 1 frequency between 250 and 500 Hz and of ≥80 dB in frequencies above 1000 Hz, (4) have had stable hearing loss for at least the past two years, and lastly (5) pass a psychological examination ensuring they had realistic expectations about the potential benefits of receiving a cochlear implant and/or using EAS (Table 1). All subjects underwent pure tone audiometry (PTA) and speech tests, pre- and postoperatively.

### 2.2. Surgical Technique

We used the same surgical technique on all subjects. The technique, which we have named the UNICAMP approach, is a mastoidectomy approach. It is based on techniques developed in various otology centers. 

### 2.3. Description of Surgical Technique

Patients were under general anesthesia, tracheal intubation, and placed in a supine position with their head turned to the contralateral side. The operative field was prepared through extensive shaving, cleaned with chlorhexidine 2%, and the attachment of the electrodes to monitor CN VII.

We used a micropore to isolate the operative field from the rest of the scalp and gave prophylaxis with cefazolin (50 mg/kg) intravenously during induction of anesthesia.

1. The main landmarks are marked: tip of the mastoid, temporal line, retroauricular incision line, area of the internal component, and area of the microphone with the help of an implant template;
2. antisepsis with 0.2% aqueous chlorhexidine, placement of sterile drapes and steri-drape 2;
3. rectilinear retroauricular incision and dissection along anatomical planes; preparation of a “cross” Palva flap (periosteal muscle) raising the four segments of the flap over the subperiosteal plane;
4. removal of small fragments of fascia and temporal muscle to occlude the cochleostomy;
5. simple mastoidectomy, identifying the lateral semicircular canal, the short ramus of the anvil, the posterior wall of the outer ear canal, the tegmen timpani, and the lateral sinus; gathering a small amount of bone dust;
6. thinning of the posterior wall of the outer ear canal, posterior tympanotomy, preservation of the incus buttress;
7. preparation of the receiver bed for the implant on the squamous portion of the temporal bone (well) using a specific implant template;
8. irrigation of the cavity with povidone-iodine (10% povidone-iodine/1% active iodine) for two minutes followed by abundant irrigation with lactated Ringer's solution;
9. irrigation of the cavity with ciprofloxacin (4 mg/mL) for two minutes followed by irrigation with lactated Ringer's solution;
10. intravenous administration of dexamethasone (8 mg) before approaching the inner ear via a cochleostomy or through the round window;
11. application of topical triamcinolone (40 mg/mL) over the round window;
12. opening the membrane of the round window; if this approach is impossible, the endosteum is opened by means of a cochleostomy;
13. positioning of the implant into the prior drilled bed;
14. Preparation of the fascia graft; making a pinhole central orifice to allow the electrode to pass snugly to be placed in the cochleostomy/round window site;
15. insertion of the electrode slowly and continuously during three minutes;
16. positioning the muscle graft around the electrode to seal the cochleostomy; placing bone dust to close the posterior tympanotomy;
17. positioning the ground electrode under the muscle-periosteum flap;
18. closure with Vicryl 3.0 sutures on the Palva flap planes and subcutaneous tissue; skin closure with Nylon 4.0;
19. cleaning of the patient and placing an external compressive dressing;
20. impedance testing, neural response telemetry (NRT), and a transorbital incidence radiograph are done to confirm the position of the intracochlear electrode.

The same surgeon and surgical team performed all six surgeries.

### 2.4. Audiometric Testing

**Figure 1:**
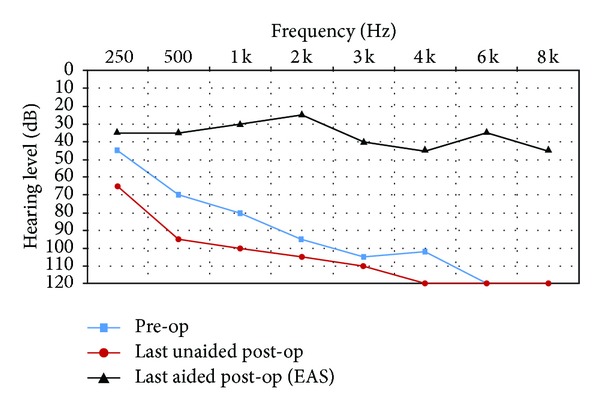
Audiometric results for Subject 1.

**Figure 2:**
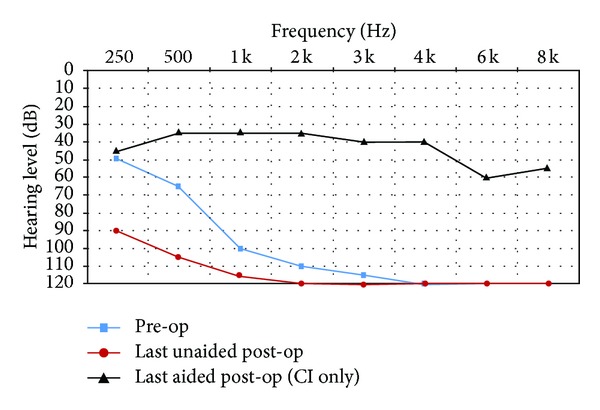
Audiometric results for Subject 2.

**Figure 3:**
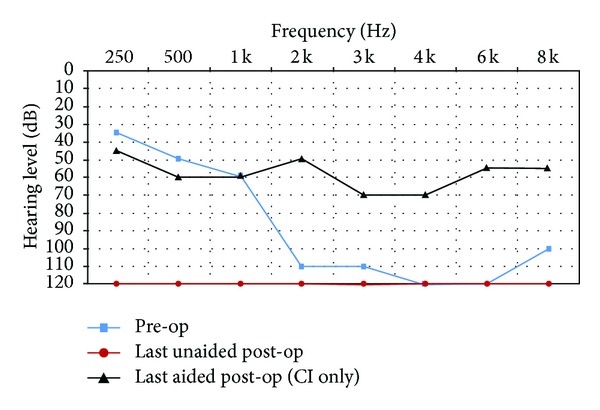
Audiometric results for Subject 3.

**Figure 4:**
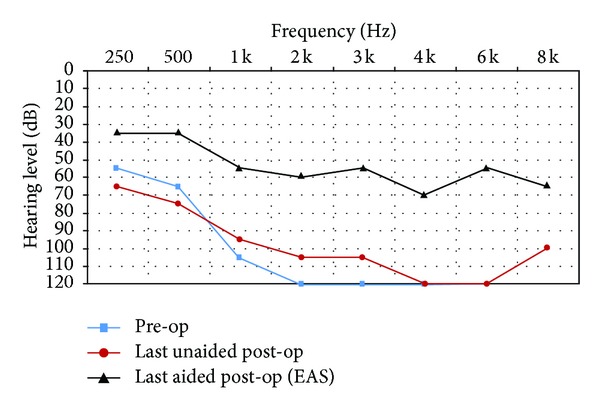
Audiometric results for Subject 4.

**Figure 5:**
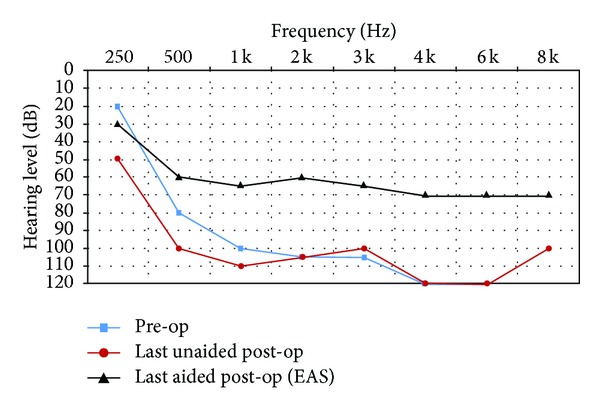
Audiometric results for Subject 5.

**Figure 6:**
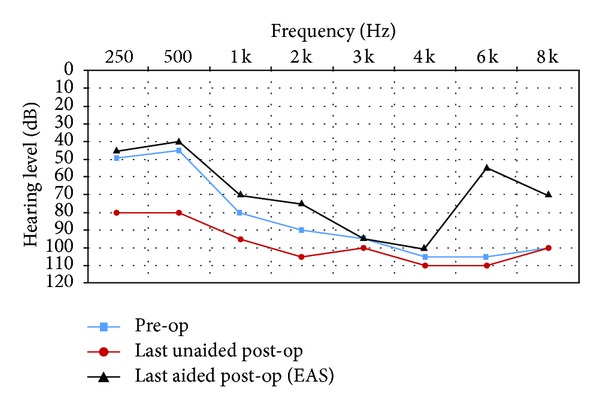
Audiometric results for Subject 6.

All subjects had unaided pure-tone audiometry tests at 250, 500, 1000, 2000, 3000, 4000, 6000, and 8000 Hz (Figures 1–6). We used an AC30-SD25 audiometer, calibrated according to ISO 389/64.

To determine subjects' residual hearing, we repeated the unaided pure-tone audiometry tests at 250, 500, 1000, 2000, 3000, 4000, 6000, and 8000 Hz three times: (1) at activation, (2) 6.5–7 months after activation, and (3) approximately 7 months after their previous test. We defined “residual hearing preservation” in three ways:

1. “total hearing preservation”: a postoperative unaided hearing loss of 0–10 dBs,
2. “partial hearing preservation”: a postoperative unaided hearing loss of >10 dB but leaving the subject with ≤80 dB hearing or better in at least one frequency between 250 and 1000 Hz,
3. “hearing preservation failure”: subject will not benefit from EAS because their unaided postoperative thresholds are >80 dB.

Lastly, to measure efficacy, all subjects had free field warble tone tests with EAS or CI-only (depending on their residual hearing) at the same postoperative intervals as were their unaided tests.

### 2.5. Speech Perception Tests

Preoperatively, all subjects took a speech perception test the same day as their implantation. We used a speech perception sentence test based on one developed by Bevilacqua et al. from several English language tests [23]. Subjects did the test with their hearing aids on, in a quiet place.

Postoperatively, all subjects repeated the speech perception test after at least 1 year of CI experience. Tests were done in subject's best-aided condition: EAS or CI-only, depending on their postoperative residual hearing. The same audiologist conducted all the pre- and postoperative tests.

### 2.6. Subjective Ratings

When the subjects did their postoperative speech tests they were asked to rate the quality of their experience with EAS/a CI over the past year on a Likert scale scored 0 to 10. A score of 0 indicated the user regretted the intervention, would not recommend it to others, and felt he/she had been better off in the past with their hearing aids. A score of 10 indicated the user was completely satisfied with the intervention and would strongly recommend it.

### 2.7. Ethics

The institutional review board approved this study and all subjects gave written informed consent.

## 3. Results

**Figure 7:**
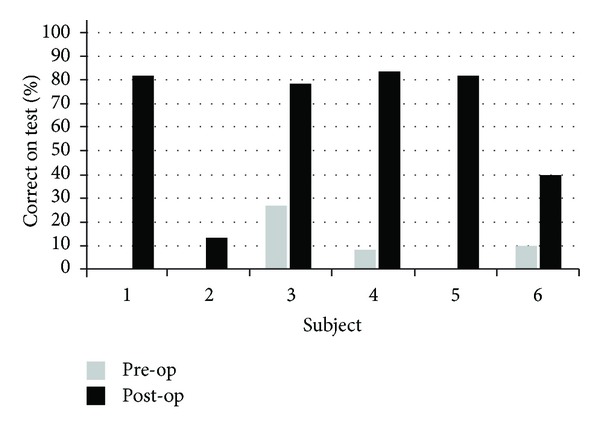
Pre- and Postoperative Speech Perception Scores. Note: Subjects 1, 2, and 5 and 0% preoperative scores. *Median speech test results pre-op: 4.0% (range 0% to 28%). **Median speech test results post-op: 80.0% (range 16% to 84%).

All surgeries were uneventful. Although we planned to implant all subjects via their round window, we could not visualize subject 2's round window and had to implant via cochleostomy. All subjects received an implant with a MED-EL FLEX^EAS^ electrode. At no frequency or test interval did any subject's aided or unaided PTA score vary by more than 10 dB from their scores at the same frequency in either of the other 2 tests. For the sake of convenience, the graphs show their PTA scores at their last postoperative test (see Tables 2, 3, and Figure 7).

All subjects' speech perception was much better after implantation (see Figure 7).

### 3.1. Subject 1

He had suffered from idiopathic hearing loss for 5 years and had been using hearing aids for 2 years without benefit. He had also been treated with corticosteroids but experienced little improvement. He was fit with EAS with the CI cut-off frequency set at 500 Hz. His preoperative tinnitus was not eliminated by surgery but is now, according to the subject, no longer bothersome. 

As you can see from his pre- and postoperative hearing test results, he derived real benefit from implantation. We achieved partial hearing preservation.

His speech perception test score improved from 0% preoperatively to 82% (with EAS) after 14 months CI experience.

He rated the quality of his experience a 9 on Likert scale, indicating he was very pleased with the intervention.

### 3.2. Subject 2

He had suffered from idiopathic hearing loss for 10 years and had been using hearing aids for 8 months without benefit. Unlike the other 5 subjects, he was implanted via cochleostomy instead of round window because we could not visualize the round window. The subject was fit with a CI only.

As you can see from his pre- and postoperative hearing test results, he derived real benefit from implantation. We, however, failed to preserve his residual hearing.

His speech test score improved from 0% preoperatively to 16% (with CI-only) after 14 months CI experience. We attribute this relatively poor speaking perception score to the fact that he (1) lost (or had stolen) his external component and so was without CI experience for approximately 11 of the 14.5 months between his activation and post-op speech test and (2) he missed audiological rehabilitation sessions.

He rated the quality of his experience a 6 on the Likert scale, indicating he was mildly pleased with the intervention.

### 3.3. Subject 3

She had suffered from idiopathic hearing loss for 10 years and had been using hearing aids for the past 6 years, with little benefit. Her preoperative tinnitus and dizziness were eliminated postoperatively. The subject was fit with a CI only.

As you can see from her pre- and postoperative hearing test results, she derived real benefit from implantation. We, however, failed to preserve her residual hearing.

Her speech test score improved from 28% preoperatively to 78% (with CI-only) after 13 months CI experience.

She rated the quality of her experience a 9 on the Likert scale, indicating she was very pleased with the intervention.

### 3.4. Subject 4

He had suffered from idiopathic hearing loss for 8 years and had been using hearing aids for 4 years in his right ear and 1 year in his left ear, with little benefit. He had also been treated with corticosteroids, with little improvement. The subject was fit with EAS with the CI cut-off frequency set at 700 Hz. His preoperative tinnitus and dizziness were eliminated postoperatively. 

As you can see from his pre- and post-op hearing test results, he derived real benefit from implantation. We achieved total residual hearing preservation.

His speech test score improved from 8% preoperatively to 84% (with EAS on) after 15 months CI experience.

He rated the quality of his experience a 10 on the Likert scale, indicating he was extremely pleased with the intervention.

### 3.5. Subject 5

He had suffered idiopathic hearing loss for 15 years and had using hearing aids for 5 years, with little benefit. The subject was fit with EAS with the CI cut-off frequency set to 350 Hz. His preoperative tinnitus was eliminated postoperatively.

As you can see from the pre- and postoperative data, he derived real benefit from implantation. We achieved partial residual hearing preservation.

His speech test score improved from 0% preoperatively to 82% (with EAS on) after 12 months CI experience.

He rated the quality of his experience a 10 on the Likert scale, indicating he was extremely pleased with the intervention.

### 3.6. Subject 6

He had suffered from idiopathic hearing loss for 15 years and had been using hearing aids for 5 years, with little benefit. The subject was fit with EAS with the CI cut-off frequency set to 1 kHz.

As you can see from the pre- and postoperative hearing test results, he derived some benefit from implantation. We achieved partial residual hearing preservation.

His speech test score improved from 10% preoperatively to 40% (with EAS) after 13 months CI experience. Despite his relatively poor postoperative speech perception score, the audiologist reports that he is improving.

He rated the quality of his experience an 8 on the Likert scale, indicating he was very pleased with the intervention.

Some benefits of the good outcomes are represented by the individual speech tests of all subjects (Tables 2, 3, and Figure 7).

## 4. Discussion

Of our 6 subjects, 1 had total residual hearing preservation, 3 had partial residual hearing preservation, and 2 had total residual hearing loss: a residual hearing preservation success rate of 4/6. Looked at another way, postoperatively, 4 subjects will benefit from EAS and 2 subjects (2 and 3) are no longer partially deaf and thus no longer EAS candidates, although they enjoy better hearing from their CI than they had had from their HA before implantation. All subjects who actually had 1-year implant experience had greatly improved postoperative speech perception scores. 3 EAS users (subjects 1, 4, and 5) scored between 82% and 84%, similar to the results of previous studies [8, 24]. Subject 6 scored poorer, only 40% but is said to be improving. Subject 3, who was fitted CI only, improved from 28% pre-op to 78% after 13 months CI experience. 

We demonstrated that cochlear implant surgery done with the aim of preserving residual hearing is highly beneficial to the hearing lives of the partially deaf—as evidenced in their extremely positive Likert scale responses—even when we fail to preserve their residual hearing. We, nonetheless, fell short of our lofty goal of 100% residual hearing preservation. We attribute this to 2 primary causes. Firstly, living in and working in Brazil, we have had very few EAS cases and less experience with hearing preservation surgery than do the surgeons whose results are featured in other articles. EAS was developed in Germany as recently as 1999 and, correspondingly, most experts come from Central Europe (Gstoettner, von Ilberg, Lenarz, Skarzynski to name a few). 

We (UNICAMP) are the only team in Brazil—a nation of almost 200 million—that does hearing preservation surgeries like this. The surgical technique is difficult and we are confident that with experience, and better-suited cases, we will improve our success rate. Our results should be seen in this context: a regional beginning.

Secondly, we had limited access to suitable EAS candidates. All subjects had idiopathic hearing loss and not all of them were “true” EAS candidates. Subjects 1 and 5 had preoperative scores of 70 dB and 80 db, respectively, at 500 Hz, whereas the maximum indication for EAS is 60 dB at 500 Hz. We implanted them anyway—as other surgical teams have done [25, 26]—because they could still benefit from EAS. If they had been “true” EAS candidates, their postoperative hearing losses of 20–25 dB at 500 Hz might have appeared less severe. 

Our residual hearing preservation results were below those of other similar studies. Skarzynski et al. [27] partially or totally preserved the hearing of 39/42 at 3 months and from 34/40 to 36/40 at 13 months after surgery. They used standard or FLEX^SOFT^ electrodes and the hearing preservation round window technique they developed and described in 2007 [15]. Arnoldner et al., achieved 11/11 residual hearing preservation after a mean follow-up of 7.85 months. He used a round window or promontorium technique and FLEX^EAS^ electrodes [16]. Gstoettner et al. reported a success rate of 15/18 after up to 12 months after EAS fitting [8]. They used the Frankfurt surgical technique and MED-EL M-electrode.

Additionally, the provision of a CI decreased subjects' tinnitus and dizziness. 4 subjects (1, 3, 4, and 5) suffered from preoperative tinnitus. Postoperatively, only subject 1 still had tinnitus, but he said it was “not too bad.” These results are consistent with past studies [28–31], which found that CI implantation usually eliminates or reduces tinnitus. Subjects 3 and 4's preoperative dizziness disappeared after surgery.

## 5. Conclusion

We strongly believe that EAS has an important place in the future of otology. While it is entering its teenage years in Central Europe, it is still in its infancy in Brazil. We hope our results will raise the profile of EAS here and hearing preservation surgery and help make it more common. Hopefully, with more experience and sufficient subjects, we—and other new teams—will soon be reporting residual hearing preservation results comparable to those of currently well-established surgeons.

## Figures and Tables

**Table 1:** Subject demographics.

Subject number	Age at implantation (years)	Sex	Duration of hearing loss (years)	Insertion
1	63	M	5	Round window
2	62	M	10	Cochleostomy
3	40	F	10	Round window
4	29	M	8	Round window
5	42	M	20	Round window
6	46	M	15	Round window

**Table 2:** PTA tests of all subjects at all intervals: unaided.

Who	Dates (days since previous test)	250 Hz	500 Hz	1 kHz	2 kHz	3 kHz	4 kHz	6 kHz	8 kHz
Subject 1	Preoperative: 01.06.2010	45	70	80	95	105	120	120	120
	Activation: 22.09.2010	65	90	105	105	105	120	120	120
	Post-op 2: 05.04.2011 (195)	65	95	100	105	110	120	120	120
	Post op-3: 14.12.2011 (253)	65	95	100	105	110	120	120	120

Subject 2	Preoperative: 21.07.2010	50	65	100	110	115	120	120	120
	Activation: 22.09.2010	90	105	115	120	120	120	120	120
	Post-op 2: 13.04.2011 (203)	90	100	110	120	120	120	120	120
	Post op-3: 07.12.2011 (238)	90	105	115	120	120	120	120	120

Subject 3	Preoperative: 22.09.2010	35	50	60	110	110	120	120	100
	Activation: 13.12.2010	115	120	120	120	120	120	120	120
	Post-op 2: 30.06.2011 (198)	120	120	120	120	120	120	120	120
	Post op-3: 11.01.2012 (195)	120	120	120	120	120	120	120	120

Subject 4	Preoperative: 26.04.2011	55	65	105	120	120	120	120	100
	Activation: 17.06.2011	65	75	95	105	120	120	120	120
	Post-op 2: 11.01.2012 (208)	65	70	90	105	120	120	120	120
	Post op-3: 18.09.2012 (251)	65	75	95	105	120	120	120	120

Subject 5	Preoperative: 11.10.2011	20	80	100	105	105	120	120	120
	Activation: 13.12.2011	50	100	110	105	105	120	120	100
	Post-op 2: 25.06.2012 (195)	50	105	110	105	110	120	120	100
	Post op-3: 19.12.2012 (177)	50	100	110	105	105	120	120	100

Subject 6	Preoperative: 18.10.2011	50	45	80	90	95	105	105	100
	Activation: 13.12.2011	80	80	95	105	105	110	110	100
	Post-op 2: 18.07.2012 (218)	85	80	90	105	105	110	105	100
	Post op-3: 08.01.2013 (174)	80	80	95	105	105	110	110	100

**Table 3:** PTA tests of all subjects at all intervals: aided (EAS or CI on).

Who	Dates (days since previous test)	250 Hz	500 Hz	1 kHz	2 kHz	3 kHz	4 kHz	6 kHz	8 kHz
Subject 1	Activation: 22.09.2010	35	35	30	25	40	35	35	45
	Post-op 2: 05.04.2011 (195)	30	35	35	20	40	30	30	50
	Post op-3: 14.12.2011 (253)	35	35	30	25	40	35	35	45

Subject 2	Activation: 22.09.2010	45	35	35	35	40	40	60	55
	Post-op 2: 13.04.2011 (203)	40	40	35	40	40	40	55	50
	Post op-3: 07.12.2011 (238)	45	35	35	35	40	40	60	55

Subject 3	Activation: 13.12.2010	45	60	60	50	40	70	55	55
	Post-op 2: 30.06.2011 (198)	40	60	55	50	45	80	65	55
	Post op-3: 11.01.2012 (195)	45	60	60	50	40	70	55	55

Subject 4	Activation: 17.06.2011	55	35	55	60	40	60	55	65
	Post-op 2: 11.01.2012 (208)	65	25	55	65	40	60	55	60
	Post op-3: 18.09.2012 (251)	55	35	55	60	40	70	55	65

Subject 5	Activation: 13.12.2011	30	60	65	60	60	70	55	70
	Post-op 2: 25.06.2012 (195)	30	60	60	55	60	70	60	70
	Post op-3: 19.12.2012 (177)	30	60	65	60	65	70	55	70

Subject 6	Activation: 13.12.2011	45	40	70	75	95	100	55	70
	Post-op 2: 18.07.2012 (218)	40	40	65	75	100	110	55	70
	Post op-3: 08.01.2013 (174)	45	40	70	75	95	100	55	70
